# circRNAs as Epigenetic Regulators of Integrity in Blood–Brain Barrier Architecture: Mechanisms and Therapeutic Strategies in Multiple Sclerosis

**DOI:** 10.3390/cells13161316

**Published:** 2024-08-06

**Authors:** Elisabetta D’Aversa, Francesca Salvatori, Mauro Vaccarezza, Bianca Antonica, Miriana Grisafi, Ajay Vikram Singh, Paola Secchiero, Giorgio Zauli, Veronica Tisato, Donato Gemmati

**Affiliations:** 1 Department of Translational Medicine, University of Ferrara, 44121 Ferrara, Italy; 2 Curtin Medical School & Curtin Health Innovation Research Institute (CHIRI), Faculty of Health Sciences, Curtin University, Bentley, WA 6102, Australia; 3 Department of Chemical and Product Safety, German Federal Institute for Risk Assessment (BfR), 10589 Berlin, Germany; 4 Research Department, King Khaled Eye Specialistic Hospital, Riyadh 11462, Saudi Arabia; 5 University Strategic Centre for Studies on Gender Medicine, University of Ferrara, 44121 Ferrara, Italy; 6 LTTA Centre, University of Ferrara, 44121 Ferrara, Italy; 7 Centre Haemostasis & Thrombosis, University of Ferrara, 44121 Ferrara, Italy

**Keywords:** multiple sclerosis, blood–brain barrier, cytoskeleton proteins, circularRNAs

## Abstract

Multiple sclerosis (MS) is a chronic inflammatory neurodegenerative disease leading to progressive demyelination and neuronal loss, with extensive neurological symptoms. As one of the most widespread neurodegenerative disorders, with an age onset of about 30 years, it turns out to be a socio-health and economic issue, thus necessitating therapeutic interventions currently unavailable. Loss of integrity in the blood–brain barrier (BBB) is one of the distinct MS hallmarks. Brain homeostasis is ensured by an endothelial cell-based monolayer at the interface between the central nervous system (CNS) and systemic bloodstream, acting as a selective barrier. MS results in enhanced barrier permeability, mainly due to the breakdown of tight (TJs) and adherens junctions (AJs) between endothelial cells. Specifically, proinflammatory mediator release causes failure in cytoplasmic exposure of junctions, resulting in compromised BBB integrity that enables blood cells to cross the barrier, establishing iron deposition and neuronal impairment. Cells with a compromised cytoskeletal protein network, fiber reorganization, and discontinuous junction structure can occur, resulting in BBB dysfunction. Recent investigations on spatial transcriptomics have proven circularRNAs (circRNAs) to be powerful multi-functional molecules able to epigenetically regulate transcription and structurally support proteins. In the present review, we provide an overview of the recent role ascribed to circRNAs in maintaining BBB integrity/permeability via cytoskeletal stability. Increased knowledge of the mechanisms responsible for impairment and circRNA’s role in driving BBB damage and dysfunction might be helpful for the recognition of novel therapeutic targets to overcome BBB damage and unrestrained neurodegeneration.

## 1. Introduction

Multiple sclerosis (MS) is a chronic progressive inflammatory demyelinating disorder of the central nervous system (CNS), leading to severe physical and cognitive disabilities as well as neurological defects [1,2,3,4]. Cognitive impairment is characterized by high variability between patients, including deficits in learning, memory, executive functions, and visuospatial processing [5]. With an age onset of about 30 years, MS is the most common neurodegenerative disease afflicting young adults [6,7]. This condition affects females more than males (sex ratio 2.5:1), with a prevalence of 120 per 100,000 individuals, depending on geographic area [8]. MS etiology remains elusive; however, it can be considered a complex multifactorial disease due to genetic predisposition in combination with hormonal and environmental factors [2,9]. Altogether, these risk factors trigger demyelinating plaques, responsible for the infiltration of proinflammatory T-lymphocytes and macrophages into the CNS across an ineffective blood–brain barrier (BBB), which results in neuronal damage and cognitive impairment [10,11].

One of the notable aspects of MS is the disruption of the BBB, a selective and dynamic endothelial cell monolayer at the interface between the vasculature and the CNS, which ensures the interflow of biological molecules and protects the brain from toxins, microorganisms, and other insults [12]. The BBB physiological functions are ensured by the presence of tight junctions (TJs) and adherens junctions (AJs) between endothelial cells, supported and controlled by cytoskeletal proteins (actin microfilaments, microtubules, and intermediate filaments [12,13]), which preserve homeostasis within the brain microenvironment by tightly regulating paracellular permeability [14].

The rearrangement and destruction of cytoskeletal architecture and cell junctions [12,15], together with the over-activation of the immune system [16], result in BBB integrity loss and subsequent increase in permeability [17], responsible for the initiation and propagation of MS active lesions. Therefore, the opportunity to act on cytoskeletal proteins and fine-tune their levels, restoring the highly complex and regulated organization and their functions, may limit BBB hyperpermeability in MS, as well as other neurodegenerative disorders such as amyotrophic lateral sclerosis, Alzheimer disease, and epilepsy [18,19,20].

In this regard, the main purpose of this review is to provide an overview of current knowledge of circularRNA (circRNA) function and their involvement in cytoskeleton regulation and stability. In fact, over the last decades, exponential advances in transcriptomics have allowed new discoveries in the multilayer regulatory role of circRNAs, a rising class of non-coding RNAs with unique characteristics and several biological functions [21]. CircRNAs, by acting directly on AJs and TJs or by indirectly regulating upstream signal pathways and transcription factors [22,23], could be considered promising drug targets to restore BBB architecture. This could aid in designing novel potential therapeutic strategies for MS in an attempt to improve the life quality and expectancy in MS patients [6,24].

## 2. Blood–Brain Barrier (BBB) Structure

**Figure 1:**
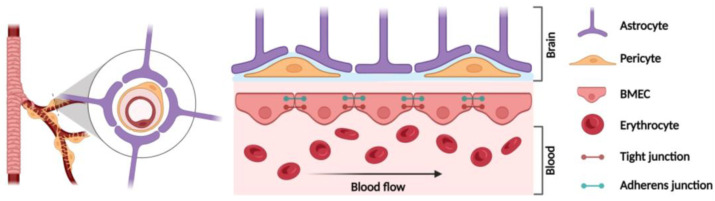
Schematic representation of BBB structure. As a part of the NVU, the cell-based BBB consists of a monolayer of BMECs that lines the blood flow, supported by brain microvascular pericytes and perivascular astrocytes. TJs and AJs between BMECs strictly limit BBB permeability [created with BioRender.com, accessed on 2 August 2024].

A healthy brain is protected by the BBB, a very specialized and selective barrier system formed by a monolayer of brain microvascular endothelial cells (BMECs), which line cerebral blood flow, as well as brain microvascular pericytes, their basal lamina, and perivascular astrocytes [25,26] (Figure 1). The barrier controls the entry and the outflow of biological molecules essential for brain metabolism and neuronal functions [12,27]. As a unique part of the neurovascular unit (NVU), the BBB has a crucial role in maintaining brain homeostasis [11] and in protecting the CNS from toxins and pathogen entry [28]. Hence, functional and structural BBB integrity is pivotal to ensure and preserve the brain microenvironment, mediating CNS communication with the rest of the body [11,12].

BMECs are specialized neuronal endothelial cells that strictly limit the movement of substances from blood to CNS, reducing the paracellular flow of hydrophilic molecules via the formation of large numbers of TJs and AJs with adjacent BMECs [12,26] (Figure 1). TJs are localized along the lateral cell membrane to completely block the cleft between cells. Structurally, the complex is composed of transmembrane adhesion proteins, which physically interact with their counterparts on the membranes of adjacent cells and simultaneously connect to the intracellular actin cytoskeleton [14,29]. The TJ transmembrane proteins include the integral membrane proteins (e.g., occludins; claudins; ZO-1), an IgG protein type, and the junctional adhesion molecule (JAM) [14]. Claudins are the main sealing components of TJs, among which claudin-5, the principal constitutive claudin at the BBB [30,31], has the function of limiting paracellular diffusion of molecules larger than 400 Da across the BBB [31,32]. Occludin interacts with scaffold cytoskeleton proteins, contributing to TJ stabilization [33,34]. In fact, in occludin-deficient mice, neurological functions are compromised due to the hyperpermeability of BBB [35]. JAMs interact with cytoskeleton proteins to recruit and assemble signaling complexes, as key regulators of barrier function. One of the roles of JAMs in the TJs of endothelial cells is the regulation of the transendothelial migration of leukocytes to inflammation sites [36].

AJs have a similar organization to TJs. They are formed by the transmembrane proteins, cadherins, responsible for the adhesion of BMECs to cytoskeletal proteins, and catenins, Ca^2+^-dependent transmembrane proteins involved in supporting cadherin association and regulating out-in signaling processes. V-cadherin is the major transmembrane AJ protein of the BBB [37]. Specifically, catenins are important for the adhesive properties of V-cadherin and for binding actin, controlling BBB leakiness [14].

The other BBB cell types support the barrier structure and enhance its functionality. In detail, microvascular pericytes are essential constituents of brain capillaries and share a basal lamina with BMECs [38,39]. Perivascular astrocytes, as constituents of neuroglia, represent the most abundant cells in the CNS and have several functions, including compartmentalization of the neural parenchyma, ionic homeostatic maintenance of the extracellular space, neurotransmitter uptake, and signaling from neurons to the vasculature [40]. Astrocytes play a critical role in BBB formation and its functional maintenance by providing secreted factors that lead to adequate association between cells of the BBB, and to the formation of strong TJs [41].

### BMEC Cytoskeleton

As previously reported, BMECs play multiple roles, including maintaining a flat endothelial morphology and retaining appropriate barrier activity through TJs and AJs [27]. Cytoskeletal proteins are required for all these functions. Similar to other cells, the cytoskeleton of BMECs consists of three filamentous proteins in constant communication with each other: actin microfilaments, microtubules, and intermediate filaments [12,13].

**Figure 2:**
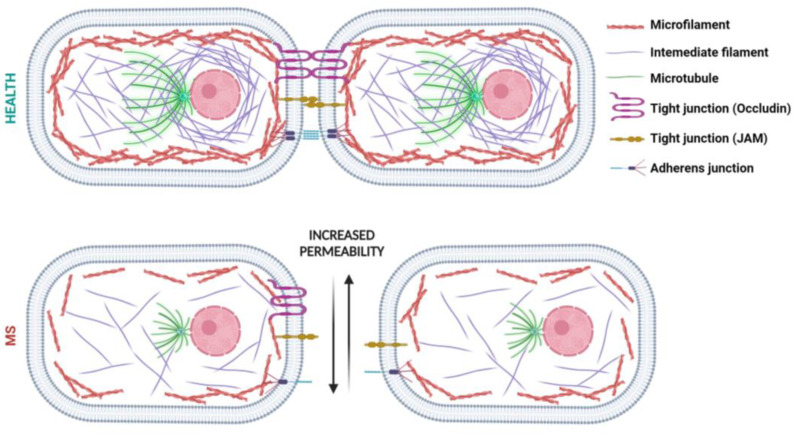
Schematic representation of cytoskeleton proteins in healthy and MS BMECs. In healthy conditions, the cortical ring of actin microfilaments supports and stabilizes the cell membrane outwardly, and joins cell–cell TJ (e.g., occludin; JAM) and AJ complexes. MTs, mainly located and packed in the center of the cell, interact with actin microfilaments directly or indirectly through IFs which are located throughout the cytoplasm, with a higher density closer to the nucleus, providing mechanical support and integrity to the cells. In MS, the complex network of IFs and MTs is demolished, along with a loss of actin microfilament organization. Consequently, a breakdown of TJs and AJs occurs, and gaps arise in the intracellular connections, resulting in increased BBB permeability [created with BioRender.com, accessed on 2 August 2024].

Microfilament proteins are found in endothelial cells as microfilamentous actin (F-Actin) [42]. In the presence of cellular stimuli, actin quickly polymerizes or depolymerizes to perform the required cellular function via proteins that bind it and regulate its action [42,43]. In addition to structural functions, actin plays a fundamental role in cell migration and transport processes, becoming a key factor in BBB permeability [38]. In endothelial cells, actin microfilaments are organized into a ring-like structure near the plasma membrane, forming the cortical actin ring [44], acting as a mechanical stabilizer to the plasma membrane [38,44]. TJs and AJs are attached to the cortical actin ring, allowing cell–cell and cell–matrix interactions and thus permitting endothelial barrier formation and its role, as shown in Figure 2 (upper panel). Specifically, the carboxyl termini of V-cadherin in the cytosolic portion link actin filaments through actin-binding proteins, including vinculin and α-actinin, as well as intracellular anchoring molecules such as p120-catenin, α-catenin, β-catenin, and γ-catenin [38,44,45]. Thus, a barrier with low and regulated permeability requires the cortical actin ring to have a linear and continuous junction structure [12,44], essential for reducing the distance between two adjacent endothelial cells [38,44].

Microtubules (MTs) are the main components of the cytoskeleton. Unlike actin microfilaments, MTs are mainly localized and packed in the middle of the cell and become more thinly distributed closer to the membrane (Figure 2). These cytoskeleton proteins assume a radial distribution since most MTs are strongly attached to centrosomes, although there are also non-centrosomal MTs distributed in the cytosol [46].

MTs are characterized by a highly organized dynamism; in fact, they undergo rapid polymerization or depolymerization following a stimulus, which allows them to grow and shrink in size, respectively. The essential role of MTs in regulating BBB functions has emerged in recent studies, as several MT-associated proteins are involved in junctions and the actin cytoskeleton. MT dynamics closely control BBB permeability by modifying cytoskeletal organization [47].

Intermediate filaments (IFs) are the strongest elements of all three cytoskeleton proteins. Their unique structure is the reason for the high dynamicity and flexibility of IFs, as well as stress resistance [48]. They are ubiquitously spaced throughout the cytoplasm with a higher density close to the nucleus (Figure 2) [49]. The most abundant IF protein in BMECs is vimentin. It participates in cell–cell interactions, immune activation, homeostasis, and tissue repair, providing structural support and mechanical integrity to the cells. In addition to maintaining the cell shape, it plays a fundamental role in protecting the vascular cells and tissues from various mechanical insults such as shear stress and/or contractile forces, strengthening cell attachment to the matrix, and regulating actin microfilaments [50]. Therefore, the unique characteristics of the vimentin network are crucial for the integrity and physiological function of the BBB [51].

Cytoskeleton proteins represent the cement and the bricks of adjacent BMECs, and the lack of a healthy structure of the cytoskeleton network can occur in BBB dysfunctions. This can be efficiently reproduced by in vitro advanced 3D prototypes based on integrated BBB-on-chip models [52], as well as nanoengineering approaches for clinical applications that more closely reflect their in vivo properties and functions [53,54].

## 3. BBB Dysfunction in MS

The BBB impairment in the pathogenesis of MS basically has a dual effect: early onset of the immune attack, and subsequently, self-sustaining demyelination and neurodegeneration processes in the CNS [55]. The mechanisms causing BBB dysfunction and integrity loss in MS are not fully understood due to the multilayer interplay of direct and/or indirect environmental and lifestyle risk factors. They include dietary habits, gut microbiome, and cigarette smoke [56,57,58,59], as well as a complex genetic scenario with more than 230 trait loci described in genome-wide association studies (GWAS) based on single nucleotide polymorphisms (SNPs) [25,60], besides several other studies [61,62,63,64,65]. Aging and age-related comorbidities are important factors in MS development and progression, and also influence BBB integrity [25,66].

Focal BBB dysfunction occurs early in the pathogenesis of new lesions, indicating that BBB impairment is an early crucial process, together with CNS immune cell infiltration, resulting in myelin damage [67]. This suggests that early diagnosis and timely intervention are strictly necessary to limit disease progression and delay the onset of irreversible neuronal loss.

BBB disruption has several effects: (i) proinflammatory cytokine and chemokine release by CNS-resident cells and infiltrating leukocytes; (ii) influx of immunoglobulins, including autoantibodies, and activation of the complement cascade; (iii) high levels of cytotoxic iron; (iv) ion channel disruption in BMECs, leading to unregulated ion passage and consequent neuronal damage; and (v) thrombin and fibrinogen influx, leading to endothelial cell activation and downstream nitric oxide and reactive oxygen species (ROS) production, as well as downregulation of TJ and AJ molecules [55,64,68,69,70,71,72].

The occurrence of the immune-mediated proinflammatory process favors the “outside-in” theory, according to which inflammation spreads from the periphery towards the CNS. The process is followed by an “inside-out” mechanism self-sustained by the central activation of lymphocytes against self-myelin that return in the peripheral compartment to recruit more proinflammatory lymphocytes, leading to a neuroinflammation-based brain-damaging loop. Both mechanisms are linked to BBB disruption [55,69,73].

Iron overload is a critical issue in BBB impairment diseases [11,63,64]. On one side, the elevated iron levels found in the brains of patients, mainly due to the death of iron-enriched oligodendrocytes and destruction of their myelin sheaths [64], contribute to barrier cell disruption through ferroptosis, a form of iron-dependent cell death due to iron-mediated lipid peroxidation [64,74,75,76,77]. On the other side, the BBB integrity loss can be the reason for the high iron levels, no longer limiting metal ion entry into the brain from the bloodstream [78,79,80].

The iron-driven oxidative stress, together with inflammation, also underlies the mitochondrial injury and the subsequent energy failure of demyelination and neurodegeneration, as in MS [81]. In detail, BBB function and myelin production are energy-intensive processes requiring more ATP and a high number of mitochondria [82]. Hence, the mitochondrial energy imbalance is another key process of BBB dysfunction.

Meanwhile, several MS patients with progressive disease show low levels of serum iron [83], and iron deficiency results in mitochondrial dysfunction and energy failure [84], causing mitochondria distress and needing the metal to make ATP. Accordingly, cells characterized by a “respiratory deficiency” play a role in pathophysiological processes in MS, including BBB integrity loss [85].

The BBB breakdown also elicits the disruption of ion channels in BMECs, resulting in an unregulated passage of ions (e.g., Ca^2+^; Na^+^; K^+^) between the blood and CNS tissue, overall leading to neuronal damage [86]. For instance, in MS, cellular Ca^2+^-regulating systems are impaired, resulting in Ca^2+^-dependent synaptic dysfunction, impaired plasticity, and neuronal death [86,87].

Moreover, MS-associated BBB integrity loss is responsible for an influx of thrombin and fibrinogen in the CNS [78]. Accumulation of fibrinogen, converted into fibrin by the action of thrombin [88], increases the activation of microglia and endothelial cells, which induces the further progression of inflammation, oxidative stress, and neuronal injury. In addition, brain fibrinogen impairs remyelination by inhibiting differentiation of oligodendrocyte precursors [89], causing low levels of cross-linked fibrin through the reduced FXIII transglutaminase activity, and may reduce cell adhesion, migration, and endothelial barrier function [90,91,92,93].

The sum of these processes also contributes to cytoskeleton architecture breakdown, promoting stress fiber production and the related downregulation and disruption of junction molecule complexes [94], which in turn negatively affects BBB permeability, resulting in subsequent disruption of brain homoeostasis, neuronal dysfunction, and neurodegeneration [14].

### Role of the Cytoskeleton on BBB Hyperpermeability in MS

Cytoskeletal proteins may act differently where BBB dysfunction is concerned. They can directly regulate BBB permeability (paracellular flow), alter transcellular exchange, and modify endothelial metabolism [14,95].

Impaired function of the junctional proteins is driven by changes in protein expression and regulation, or post-translational modifications [14,96]. As a result of different harmful stimuli, the AJs and TJs of adjacent BMECs are damaged, causing BBB failure. Specific MS stressors, such as cytokines, chemokines, free radicals, and iron overload, cause the formation of stress fibers through aberrant actin reorganization in BMECs [97,98]. Enhanced actin activity triggers high cytoskeleton tension, resulting in increased cell spacing that causes direct hyperpermeability of the BBB. Actin reorganization can occur directly from structural changes in F-actins, resulting in the loss of the cortical ring scaffold, or from disturbances in the organization of IF and MT networks [12,97,99], as shown in Figure 2 (lower panel). In turn, the ensuing cytoskeletal disorganization elicits a cellular influx of Ca^2+^, the production of nitric oxide and ROS, and further formation of stress fibers, triggering a feedback loop capable of amplifying the negative effects on the junctions [12,94,100].

Regarding microfilaments, abnormal regulation of Wnt/β-catenin signaling is involved in the pathogenesis of several neurodegenerative diseases, including MS [101]. Hussain et al. induced endothelial knockout of β-catenin in adult mice and determined its impact on BBB permeability. The researchers noted low mRNA levels of Wnt target genes, indicating dramatic downregulation of endothelial Wnt/β-catenin signaling, leading to both paracellular and transcellular transport alteration [102]. Accordingly, knockdown of alpha-actinin leads to changes in organization of the actin cytoskeleton and loss of cell polarity [103], as occurs in MS [104].

With the aim to investigate cytoskeletal dysfunctions in MS, Nishihara et al. developed BMEC-like iPSCs-derived cells from both healthy controls and MS patients, establishing an in vitro model of BBB that preserves structure and transport characteristics. Unlike healthy BMECs, cells derived from MS patients were larger in size and showed impaired junctional integrity (particularly with V-cadherin, claudin-5, and occludin), impaired barrier properties, and impaired efflux pump activity. In addition, BMEC-like MS-derived cells showed an inflammatory phenotype with increased expression of adhesion molecules and interactions with immune cells [67].

After demonstrating ZO-1 and occludin abnormalities in the white matter of MS patients, Padden et al. described the role of JAM impairment in MS pathogenesis. In MS lesions, although high levels of JAM proteins were found, they are muddled and less organized [105]. In detail, JAM-A alterations mainly affect vessels, while high levels of JAM-B proteins were found in brain parenchyma. However, JAM alterations both affect BBB junctional tightness and leukocyte trafficking [105,106].

The role of IFs in MS endothelial permeability has not been extensively investigated, but studies have mainly involved intracellular filamentous vimentin. First, MS stressors activate various protein kinases (e.g., PKA and PKC), triggering the phosphorylation of the head domain of vimentin and its depolymerization, with consequent BMEC intercellular junction loss. More specifically, when the IF network is altered, the cell loses tensegrity and actin organization, which has significant effects on the disruption of BBB integrity and both transcellular and paracellular permeability [12,107,108]. From a structural point of view, the IFs collapse around the nucleus and redistribute [12,109], as shown in Figure 2 (lower panel).

Several studies show extensive involvement of molecules of the immune response associated with allergy in autoimmune demyelination, as for MS [110,111]. Among these stressors, high levels of histamine neurotransmitter are directly implicated in BBB dysfunction on different cellular and molecular levels [112,113]. In detail, Shasby et al. have shown that high levels of histamine stimulate phosphorylation of AJs (e.g., V-cadherin and catenin) and alter their link to vimentin, hindering in turn AJ architecture [114]. However, the direct relationship between allergies and the worsening of MS symptoms is still under investigation.

Finally, tubulin cytoskeleton dysfunctions are often described as characteristic features of neurodegenerative diseases, including MS. In normal endothelial cells, the MT system has strongly pronounced convergence in the centrosome region. Near the cell edge, MT density decreases significantly. MS is associated with disturbed MT organization due to stress fibers. The rapid depolymerization and disruption of peripheral MTs are essential early events in the complex mechanisms of endothelial barrier dysfunction. Researchers have proven the hypothesis that the entire system of MTs changes, which means that MTs grow out of the centrosome and become significantly shorter [46], as shown in Figure 2 (lower panel). In this regard, Mentor et al. demonstrated that, following treatment with Nocodazole, a MT depolymerizing agent, neuronal endothelial bEnd5 cells exhibit TJ anastomosis misalignments in adjacent cells, failing to establish a well-regulated brain–endothelial barrier [15], endorsing MTs’ indispensable role in the maintenance of regulatory permeability across the BBB.

A fine targeting and controlling of cytoskeletal protein levels may restore their complex and regulated organization. Their rebalancing could mitigate and prevent BBB hyperpermeability, the associated demyelination, and related brain damages.

## 4. Circular RNAs (circRNAs)

The exponential development of biotechnologies has enabled new discoveries in RNA biology and transcriptomics [56,115]. During the last few decades, the advancement in revolutionary new high-throughput technologies (e.g., next-generation sequencing) and bioinformatics [21] has advanced knowledge of endogenous RNA complexities and transcriptional regulation, first with the identification of microRNAs (miRs) and more recently with a newly recognized large class of non-coding RNAs (ncRNAs) known as circRNAs [116,117,118,119]. Although these species were described for the first time in the 1970s, circRNAs have attracted extensive attention in recent years because of their specific characteristics and functions [23,116,117,118]. Here, we provide an overview of circRNA features, followed by a discussion on their involvement in cytoskeleton architecture impairment.

### 4.1. CircRNAs: Structure and Functions

CircRNAs differ from other non-coding transcripts due to their unique circular secondary structure, which is created by back-splicing a single-stranded linear transcript, forming a covalent bond, resulting in a closed-loop molecule lacking polyadenylation and capping [118,119,120]. Hence, circRNAs are 2.5–5 times more stable than linear RNAs, due to the absence of exposed ends targetable by exoribonucleases, besides being highly conserved across species and displaying a high degree of cell and tissue specificity, particularly in the brain [63,120,121]. High-throughput RNA-seq studies performed in eukaryotes have identified a large number of circRNAs different in type and length, ranging from approximately 100 to 4000 base pairs [23,118,122].

**Figure 3:**
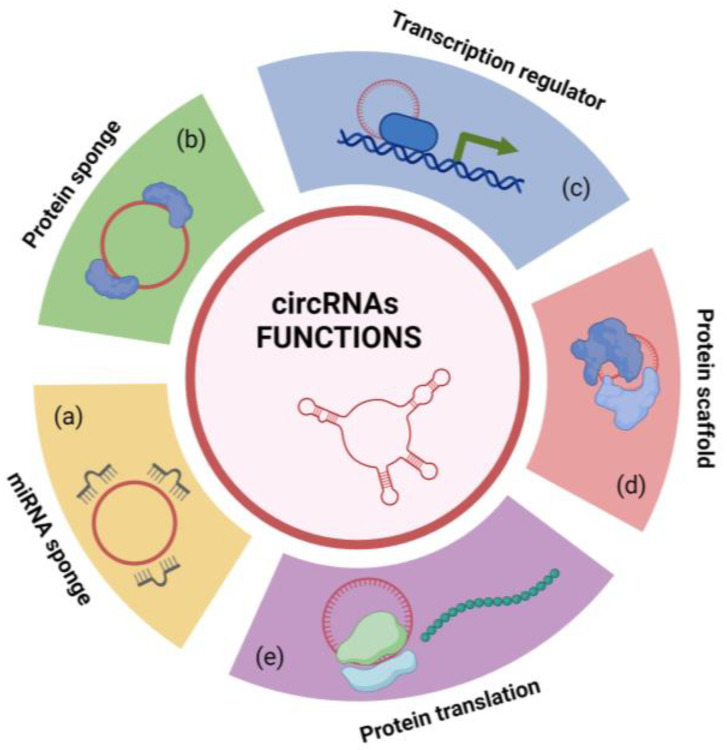
Schematic representation of circRNA functions. CircRNAs act as (**a**) miR and (**b**) protein sponges suppressing miR and protein downstream functions, (**c**) transcription regulators facilitating nuclear translocation of transcription factors, (**d**) protein scaffolds structurally supporting interactions between proteins, and (**e**) templates for the translation of small proteins [created with BioRender.com, accessed on 2 August 2024].

CircRNAs are considered molecular players with a pivotal role in controlling gene expression at transcriptional and post-transcriptional levels, acting as powerful epigenetic regulators [21,123]. Currently, several circRNA functions have been elucidated, as graphically depicted in Figure 3.

A well-established ability of circRNAs is to act as miR sponges [124] (Figure 3a). In that role, circRNAs are able to specifically sequester miRs to avoid binding to their target mRNAs, thus serving as indirect regulators of specific gene expression networks, influencing the availability and performance of miRs [23,122,125,126].

Although miR sponging is the more well-known mechanism of action, transcriptomic studies have discovered thousands of circRNAs containing a small number of miR binding sites, which suggests that the majority of circRNAs possess other functions and exert additional relevant epigenetic effects [124,127].

Certain circRNAs are able to sequester proteins or translocate them between subcellular compartments, promoting their retention in the cytoplasm and suppressing their downstream functions (Figure 3b). In contrast, other circRNAs facilitate nuclear translocation of several proteins, such as transcription factors, promoting their respective actions (Figure 3c). The mechanisms that allow circRNAs to influence protein trafficking are currently not completely known [125,127,128]. More in-depth research, together with the emergence of spatial transcriptomics [129,130], shows circRNAs as protein scaffolds able to structurally support and facilitate interactions between two or more proteins, for instance co-localizing enzymes and their substrates [131] (Figure 3d).

Despite circRNAs generally being ncRNAs, several studies have provided evidence that some of these circular molecules have the ability to translate into small proteins in a cap-independent and ribosome-entry-site (IRES)-dependent manner [132] (Figure 3e). The functions of many circRNA-derived proteins have yet to be identified, although some have been implicated in cancer progression and neurodegenerative diseases [23,133].

The fascinating description of the circRNA landscape suggests a two-faced nature in these molecules. Specifically, under physiological conditions, circRNAs play essential regulatory roles in many cellular processes, carrying out various biological functions, while when dysregulated, they are implicated in the pathogenesis of several diseases [51], such as neurodegenerative disorders, including MS [134,135,136,137,138].

Accordingly, due to their high stability, cell- and tissue-specific expression, and ability to epigenetically regulate at different levels, circRNAs are considered powerful and promising biomarkers for multiple complex diseases [125,136,139]. In addition, their presence in various body fluids, such as plasma and saliva, allows circRNA detection and analysis in a non-invasive manner, making them even more suitable as biomarkers [140]. Therefore, they are currently being explored as potential high-powered diagnostic markers and target molecules to be overexpressed or knocked down as a therapeutic approach [127,140].

Even though circRNAs have been found to be abundant in the CNS and tend to accumulate with aging, the potential employment of circRNAs as biomarkers in neurodegenerative diseases has not been well explored [118].

Changes in circRNA levels might be the direct cause of MS pathogenesis as well as the downstream effect of MS pathogenic processes, serving as dual indicative biomarkers. Actually, several studies have demonstrated that epigenetic changes and environmental (risk) factors, such as vitamin D deficiency and infections (e.g., Epstein–Barr virus), seem to influence MS susceptibility and progression acting on ncRNA expression, circRNAs included [141,142,143].

Recently, for the first time, studies have demonstrated that circRNAs play a role in BBB breakdown and consequent brain dysfunction by acting on the cytoskeleton proteins [144,145,146].

### 4.2. CircRNAs as Potential Therapeutic Targets for Cytoskeleton Architecture in BBB Integrity

The role of cytoskeleton proteins and their dynamic contribution to BBB architecture have been discussed in the previous sections. Loss of cytoskeleton homeostasis and the consequent loss of integrity of cellular focal junctions are hallmarks of BBB breakdown, resulting in enhanced barrier permeability, a distinctive feature of MS [14]. The below section aims to illustrate the available knowledge of circRNAs as modulators of dynamic cytoskeletal structures (e.g., AJs, TJs, and microtubules) and their impact on BBB integrity, which currently remains poorly investigated.

Liao et al. performed research to assess the expression profile of circRNAs in HUVEC endothelial cells. RNAseq analysis, followed by RT-qPCR validation of the discovered transcripts, allowed the identification of 16 circRNAs directly or indirectly related to AJs, listed in Table 1 [147]. For instance, five circRNAs were found to be associated with alpha-actinin1 (ACTN1), a cytoskeletal protein isoform found alongside AJs, where it is involved in the cross-linking of actin filaments and their binding to the cell membrane, thus playing a crucial role in the organization of the cytoskeleton framework as well as in the maturation of focal adhesion [148,149]. In detail, hsa_circ_ACTN1_2453, hsa_circ_0002913, and hsa_circ_0032321 negatively affect ACTN1 expression, whereas hsa_circ_ACTN1_2450 and hsa_circ_0007644 promote protein expression [147].

The same study identified the promising hsa_circ_0074158 as a potential therapeutic target, experimentally demonstrating that the knockdown molecule can significantly enhance the expression of V-cadherin, acting on catenin alpha 1 (CTNNA1) [147], a core member of the cadherin-catenin complex that contributes to the arrangement of the junction [158]. V-cadherin disruption leads to AJ breakdown, contributing to endothelial hyperpermeability [159]. In addition, hsa_circ_0074158 downregulation reverses V-cadherin suppression by reducing endothelial hyperpermeability and potentially restoring barrier protection. Consequently, high levels of hsa_circ_0074171, hsa_circ_0008194, and hsa_circ_0007440 negatively affect CTNNA1 expression, destroying barrier integrity [147], and hence potentially being therapeutically targetable.

The tyrosine phosphatase receptor type M proteins (PTPRMs) are key players in the regulation of cell-to-cell adhesions [160], particularly the AJ pathway involved in BBB function [161]. Liao et al. have found that hsa_circ_0004188, hsa_circ_0002872, and hsa_circ_0046813 negatively influence PTPRM, whereas the expressions of hsa_circ_0006114 and hsa_circ_PTPRM_4378 enhance protein function [147].

Furthermore, researchers identified hsa_circ_0008016 and hsa_circ_0005564 as positive modulators of fibroblast growth factor receptor 1 (FGFR1) [147]. This receptor system is involved in the maintenance of vascular BBB integrity via enhancing V-cadherin stabilization at AJs, even though the precise molecular mechanism is not well understood [162,163,164].

As previously discussed, “zippering up” TJs also prevents harmful substances from crossing the BBB and entering the brain [165]. Only a few studies have discovered circRNA involvement in TJ barrier regulation (Table 1). For example, hsa_circ_DLGAP4 is highly expressed in healthy CNS but significantly decreased in neurological disorders, resulting in downregulation of TJ proteins (e.g., ZO-1, claudin-5, and occludin) and associated BBB damage [150,151]. In detail, hsa_circ_DLGAP4 regulates TJ proteins by indirectly acting as an miR-143 sponge. High miR-143 levels have a deleterious effect on BBB integrity, so pathological hsa_circ_DLGAP4 downregulation leads to its failure to sponge miR-143, resulting in TJ and barrier damage [150,166,167].

Likewise, high levels of hsa_circ_USP1 ensure the proper function of ZO-1, claudin-5, and occludin, and in contrast, circRNA downregulation disrupts barrier integrity and increases its permeability by TJ-related protein reduction [152]. Bioinformatics prediction using the CircInteractome database (https://circinteractome.nia.nih.gov/, accessed on 2 August 2024), and subsequent experimental validation, reveal hsa_circ_USP1 as an miR-194-5p sponge, which in turn regulates the transcription factor FLI1 [152].

In contrast, has_circ_HECW2 expression in BMECs promotes the degradation of ZO-1, occludin, and claudin-5. In detail, has_circ_HECW2 competitively sponges miR-30d to up-regulate the expression of autophagy-related 5, which is involved in BBB damage due to TJ depletion. Hence, specific blockage of this circRNA may be envisioned as a potential therapeutic target for the treatment of BBB damage [153,154].

Again, low expression levels of has_circ_7632 and hsa_circ_Cul2 significantly decrease the expression of vimentin [155,156], the most abundant intermediate filament, which plays a key role in BBB permeability, by microtubule and actin reorganization and by direct binding to VE-cadherin or integrin proteins [12]. hsa_circ_2858, instead, acts as an endogenous RNA competitor for the miR-17 family and for miR-93-5p, promoting vascular endothelial growth factor A (VEGFA) expression in BMECs, thus leading to TJ disruption and BBB hyperpermeability [143,153,168].

Particularly noteworthy is the hsa_circ_ZNF609, a microtubule-related circRNA (Table 1). Rossi et al. explain the circRNA molecular mechanism, revealing that circZNF609, widely expressed in the brain, directly binds several mRNAs. Among them, CKAP5 is a key molecule for the stability and dynamic functions of microtubules [157]. Consequently, its downregulation could be partially responsible for cytoskeleton instability and BBB dysfunction.

In summary, the exploration of epigenetic mechanisms that preserve cytoskeleton architecture and ECM dynamics, paves the way for novel attractive circRNA-based therapeutic approaches. Strategies aiming at protecting and preserving BBB integrity and/or reverse endothelial dysfunction may have a great therapeutic potential not only in classical neurodegenerative diseases but also in other complex diseases in which epigenetic mechanisms and dysfunctional endothelial cells play a role [169,170,171].

## 5. Conclusions, Remarks and Future Directions

Individuals diagnosed with multiple sclerosis (MS) often deal with a diminished quality of life due to physical and cognitive impairments that can significantly impact their daily activities and overall well-being [22]. MS is a prevalent chronic inflammatory neurodegenerative condition leading to a range of debilitating symptoms and challenges for those living with the disease [1,2,9]. Interestingly, recent clinical studies suggest that a multilayer approach, involving physical activity and a healthy diet, may ameliorate disease progression and improve disability outcome, according to the individual genetic background and in line with the P4 medicine ideal [172]. Despite the urgent need for effective and long-lasting treatments to manage MS, such options are currently lacking, leaving patients and healthcare providers with limited options for disease management and symptom control. A key early feature of MS is the compromised integrity of the blood–brain barrier (BBB), a crucial protective barrier in the central nervous system that regulates the passage of substances between the bloodstream and the brain [76]. This disruption in the BBB’s function results in abnormal permeability, potentially allowing harmful immune cells and molecules to enter the brain and contribute to the disease progression. Addressing this distinct characteristic through targeted therapeutic strategies aimed at restoring the integrity of tight and adherens junctions between endothelial cells at the interface with the central nervous system holds promise for developing more effective treatments that could slow or halt the progression of MS and improve the quality of life for those affected by this complex and challenging condition. This review seeks to provide an overview of the significance of cytoskeleton proteins in BBB function and to explore the potential role of circular RNAs (circRNAs) in maintaining cytoskeletal homeostasis and BBB structure. CircRNAs have gained attention in recent years for their regulatory role in gene expression at multiple levels, owing to their stability and tissue-specific expression patterns [115,116,117,118]. They are increasingly recognized as valuable biomarkers for monitoring disease progression and as targets for novel therapeutic interventions [115,116,117,124].

Circular RNAs (circRNAs) have the potential to serve as promising therapeutic targets for enhancing the integrity of the BBB and consequently improving the life expectancy of individuals with MS. CircRNAs can influence the tight junctions (TJs) and adherens junctions (AJs) directly or indirectly by modulating upstream signaling pathways and transcription factors [143,145,147,152,155,156,157,167]. However, our understanding of how circRNAs impact the complex architecture of the BBB is still in its nascent stages, indicating the need for more comprehensive and detailed research in this area to determine suitable druggable targets. To advance this field, extensive high-throughput next-generation sequencing (NGS) studies, in conjunction with sophisticated bioinformatics tools and functional validation experiments, are essential for identifying and characterizing novel circRNAs capable of regulating BBB cytoskeletal proteins. Moreover, the utilization of innovative spatial transcriptomics techniques that specifically target circRNAs could greatly enhance our understanding of these complex biological processes by mapping out the spatial expression patterns of circRNAs within the intricate structural framework of tissues [128]. Despite the inherent challenges associated with this cutting-edge approach, the development of therapeutic strategies focused on circRNAs presents a promising and rapidly expanding frontier in the treatment of MS, aligning seamlessly with the ever-evolving landscape of modern medicine and offering new avenues for more effective interventions.

## Figures and Tables

**Table 1:** List of circRNAs related to the AJ and TJ pathways, TJs, and microtubules.

circRNA ID	Protein Target	Regulation	Ref.
hsa_circ_0074158	CTNNA1	Suppressor	[147]
hsa_circ_0004188	PTPRM	Suppressor	
hsa_circ_ACTN1_2453	ACTN1	Suppressor	
hsa_circ_0002872	PTPRM	Suppressor	
hsa_circ_0074171	CTNNA1	Suppressor	
hsa_circ_0046813	PTPRM	Suppressor	
hsa_circ_0002913	ACTN1	Suppressor	
hsa_circ_0032321	ACTN1	Suppressor	
hsa_circ_0008194	CTNNA1	Suppressor	
hsa_circ_0007440	CTNNA1	Suppressor	
hsa_circ_0006114	PTPRM	Enhancer	
hsa_circ_0008016	FGFR1	Enhancer	
hsa_circ_PTPRM_4378	PTPRM	Enhancer	
hsa_circ_ACTN1_2450	ACTN1	Enhancer	
hsa_circ_0007644	ACTN1	Enhancer	
hsa_circ_0005564	FGFR1	Enhancer	
hsa_circ_DLGAP4	ZO-1, claudin-5, occludin	Enhancer	[150,151]
hsa_circ_USP1	ZO-1, claudin-5, occludin	Enhancer	[152]
has_circ_HECW2	ZO-1, claudin-5, occludin	Suppressor	[153,154]
has_circ_7632	vimentin	Enhancer	[155]
hsa_circ_Cul2	vimentin	Enhancer	[156]
hsa_circ_2858	VEGFA	Enhancer	[143,153]
hsa_circ_ZNF609	CKAP5	Enhancer	[157]
